# High monocyte to lymphocyte ratio is associated with impaired protection after subcutaneous administration of BCG in a mouse model of tuberculosis

**DOI:** 10.12688/f1000research.14239.2

**Published:** 2018-06-27

**Authors:** Andrea Zelmer, Lisa Stockdale, Satria A. Prabowo, Felipe Cia, Natasha Spink, Matthew Gibb, Ayad Eddaoudi, Helen A. Fletcher

**Affiliations:** 1 London School of Hygiene and Tropical Medicine, Department of Immunology and Infection, Keppel Street, London, WC1E 7HT, UK; 2 Baylor Institute for Immunology Research, 3434 Live Oak Street, Dallas, Texas, 75204, USA; 3 UCL Great Ormond Street Institute of Child Health, 30 Guilford Street, London, WC1N 1EH, UK

**Keywords:** Tuberculosis, animal models, BCG, vaccine, ML ratio, mice

## Abstract

**Background: **The only available tuberculosis (TB) vaccine, Bacillus Calmette-Guérin (BCG), has variable efficacy. New vaccines are therefore urgently needed. Why BCG fails is incompletely understood, and the tools used for early assessment of new vaccine candidates do not account for BCG variability. Taking correlates of risk of TB disease observed in human studies and back-translating them into mice to create models of BCG variability should allow novel vaccine candidates to be tested early in animal models that are more representative of the human populations most at risk. Furthermore, this could help to elucidate the immunological mechanisms leading to BCG failure. We have chosen the monocyte to lymphocyte (ML) ratio as a correlate of risk of TB disease and have back-translated this into a mouse model.

**Methods**: Four commercially available, inbred mouse strains were chosen. We investigated their baseline ML ratio by flow cytometry; extent of BCG-mediated protection from M *ycobacterium tuberculosis* infection by experimental challenge; vaccine-induced interferon gamma (IFNγ) response by ELISPOT assay; and tissue distribution of BCG by plating tissue homogenates.

**Results:** The ML ratio varied significantly between A/J, DBA/2, C57Bl/6 and 129S2 mice. A/J mice showed the highest BCG-mediated protection and lowest ML ratio, while 129S2 mice showed the lowest protection and higher ML ratio. We also found that A/J mice had a lower antigen specific IFNγ response than 129S2 mice. BCG tissue distribution appeared higher in A/J mice, although this was not statistically significant.

**Conclusions:** These results suggest that the ML ratio has an impact on BCG-mediated protection in mice, in alignment with observations from clinical studies. A/J and 129S2 mice may therefore be useful models of BCG vaccine variability for early TB vaccine testing. We speculate that failure of BCG to protect from TB disease is linked to poor tissue distribution in a ML high immune environment.

## Introduction

Tuberculosis (TB), caused by *Mycobacterium tuberculosis* ( *Mtb*) is the leading cause of death from a single infectious agent. Multidrug-resistant TB remains a public health crisis, and only one vaccine (the *M. bovis*-derived Bacillus Calmette-Guérin, BCG) is currently licensed for clinical use. To meet the sustainable development goal of ending the TB epidemic by 2030, new treatments and vaccines are both urgently needed.

BCG efficacy is highly variable ^1, 2^. The reasons why BCG protects when it does and why it fails when it doesn’t are incompletely understood, but are crucial to the successful design and testing of new vaccines ^3^. Furthermore, to be able to accurately assess vaccine candidates in models where BCG both protects and does not protect very early on in the vaccine development pipeline would be highly advantageous and de-risk failure in later stage clinical trials. There is however a lack of a broad range of tools that can collectively predict with some confidence whether a vaccine will be protective.

One of the tools for very early testing of TB vaccine candidates is a mouse model. However, in order to obtain meaningful information, new vaccine candidates should be tested in models that are clinically relevant and reflect the breadth and heterogeneity of immune environments and varying BCG efficacy found in human populations. We propose that this could be achieved by back-translating observations from clinical studies, such as correlates of risk of TB disease, into animal models.

A number of correlates of risk of TB disease have recently been identified, including transcriptomic mRNA signatures in blood, T cell activation, and monocyte to lymphocyte (ML) ratio ^4– 8^, while others are being investigated (reviewed in 9). We have chosen the ML ratio to provide proof of principle that correlates of risk can be back-translated into the mouse to develop a model for vaccine testing that better reflects the populations most at risk of BCG failure.

In this study, we show that inbred, commercially available mouse strains have differing ML ratios, and that a high ML ratio is associated with lower BCG-mediated protection from experimental *Mtb* challenge. We further suggest that lack of BCG dissemination and/or persistence in ML high mice impairs protection.

## Methods

### Ethics statement

All animal work was carried out in accordance with the Animals (Scientific Procedures) Act 1986 under a license granted by the UK Home Office (PPL 70/8043), and approved locally by the London School of Hygiene and Tropical Medicine Animal Welfare and Ethics Review Body.

### Animals and experiment design

The following mouse strains were used for the experiments reported here (abbreviated names used throughout the manuscript are given in brackets): A/JOlaHsd (A/J); DBA/2OlaHsd (DBA/2); C57BL/6JOlaHsd (C57Bl/6); 129S2/SvHsd (129S2). Female mice were acquired from Envigo UK at 5–7 weeks of age. Animals were housed in specific pathogen-free individually vented cages with environmental enrichment (play tunnel and tapvei block), with 12 hours light / 12 hours dark cycles, at temperatures between 19° – 23°C and relative humidity of 45 – 65%. Mice were fed sterilized diet RM1 and filtered water ad libitum, and were allowed to acclimatize for at least 5 days before the start of any experimental procedure. Mice were allocated to cages as groups of 5 by technical staff not involved in experimental procedures or data analysis, and mice of the same strain were housed together. Each cage was allocated to a treatment in no particular order, but without formal randomisation. Animal welfare was assessed twice every day before and during the study.

**Figure 1.**
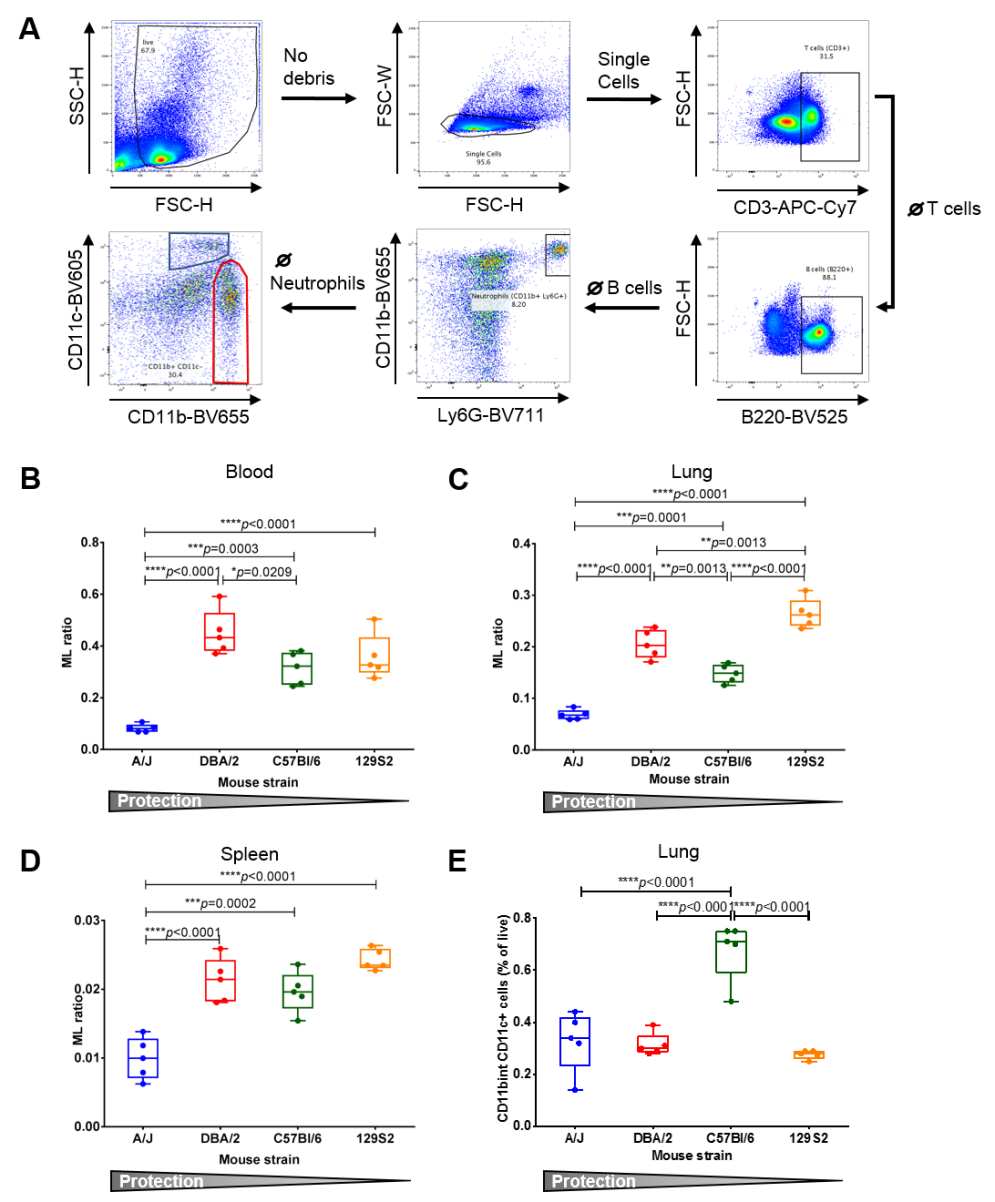
The ML ratio differs significantly in four different inbred mouse strains. **A** Gating strategy for flow cytometric analysis. Cells from naïve animals were fixed, stained and data acquired as described in Materials and Methods. Cell debris was gated out by use of a FSC-SSC gate, followed by gating on single cells (FSC-H and FSC-W). A sequential gating strategy was then applied to determine the frequency of T cells (CD3 ^+^), B cells (B220 ^+^), neutrophils (CD11b ^+^ Ly6G ^+^), monocytes/macrophages (CD11b+ CD11c ^low-int^, red gate) and CD11b ^int^ CD11c ^+^ cells (blue gate) as a percentage of single cells. Plots shown are from a sample of a C57Bl/6 spleen. **B**– **D** The ML ratio was calculated by dividing the percentage of monocytes/macrophages by the sum of the percentages of B and T cells. ML ratio was analysed in blood ( **B**), lung ( **C**) and spleen ( **D**) of four different mouse strains. **E** Percentage of CD11b ^int^ CD11c ^+^ cells in the lung of four different mouse strains, likely to be enriched in alveolar macrophages. Each symbol represents one animal; box plots represent the median (middle line), 25 ^th^ to 75 ^th^ percentile (box) and minimum to maximum value (error bars). Data sets are presented in order of decreasing protection. *p* values were determined using ordinary ANOVA with Holm-Sidak test for multiple comparisons. Multiplicity adjusted *p* values are reported. A *p* value <0.05 was considered statistically significant.

**Figure 2.**
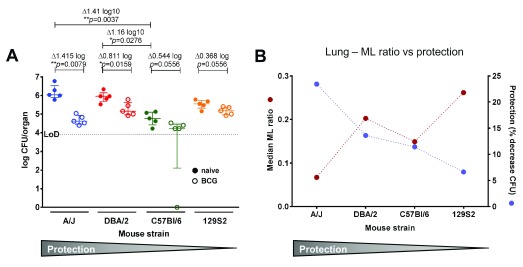
Protection from Mtb infection varies after BCG vaccination in ML high and low mouse strains. **A** Mice of each strain were immunised s.c. with 2x10e5 CFU BCG Pasteur (open circles) or left untreated (filled circle), and infected i.n. with 30 CFU Mtb Erdmann 6 weeks later. Bacterial burden in the lungs of all animals was enumerated 7 weeks after challenge. Each symbol represents one animal. Bacterial numbers are given as log10 CFU per whole organ. No bacteria were detected in one of the C57Bl/6 samples; this value was set to zero. Δ indicates the difference in bacterial burden between naïve and BCG immunised mice. *p* values were determined by Kruskal-Wallis test with Dunn’s post-test for multiple comparisons between naïve groups, and multiplicity adjusted *p* values are reported. Individual Mann-Whitney tests to compare naïve with BCG immunised groups of each mouse strain. *p*<0.05 was considered statistically significant. Error bars represent the median and interquartile range. LoD: limit of detection. **B** ML ratio in comparison to protection in the lung of ML high and low mice. The median ML ratio in lung (red; as in Figure 1) is plotted together with protection expressed as the median % decrease in CFU (blue; as in **A**).

**Figure 3.**
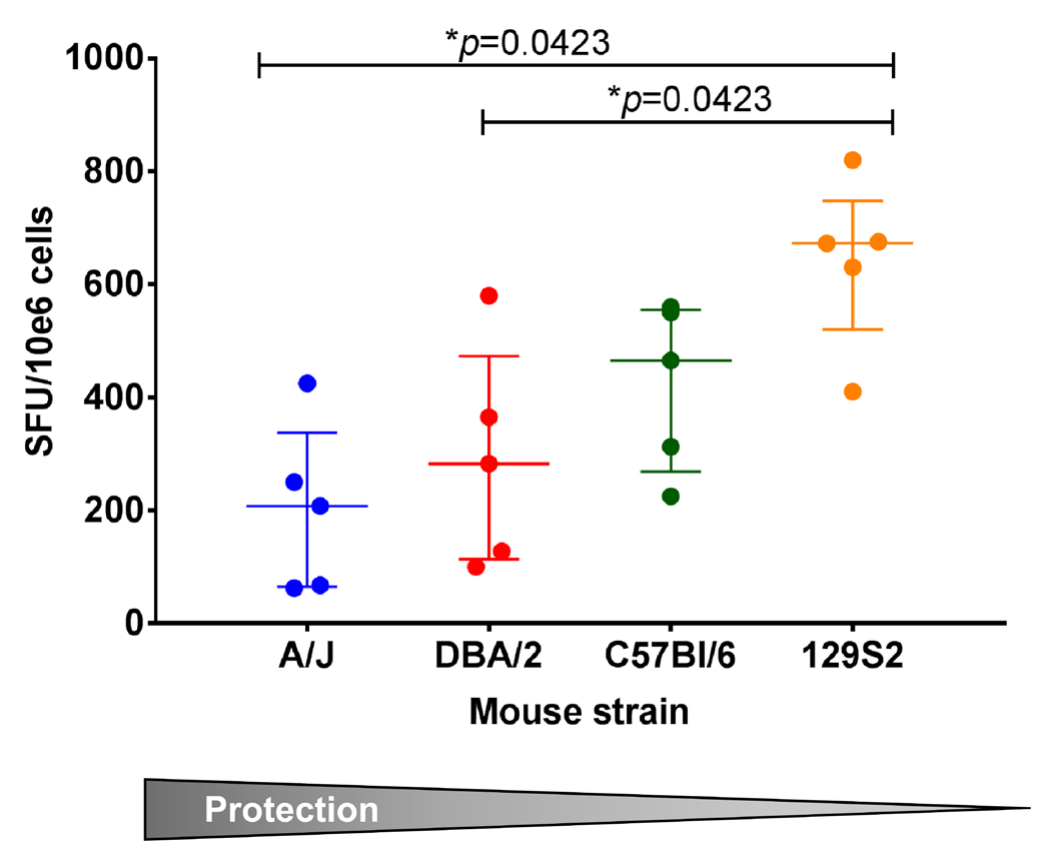
Antigen-specific interferon gamma response varies in splenocytes of ML high and ML low mouse strains. Splenocytes were isolated from BCG-immunised or control mice at the time of Mtb challenge (6 weeks after immunisation), and restimulated with PPD. The number of IFNγ producing cells were enumerated using an ELISPOT assay (presented as spot forming units [SFU] per 10e6 cells). Non-specific background measured in unstimulated duplicate wells was removed. Data sets are presented in order of decreasing protection. Each symbol represents one animal. *p* values were determined by using one-way ANOVA and Dunn’s post test for multiple comparisons. Error bars represent the median and interquartile range. Multiplicity adjusted *p* values are reported. *p*<0.05 was considered statistically significant.

**Figure 4.**
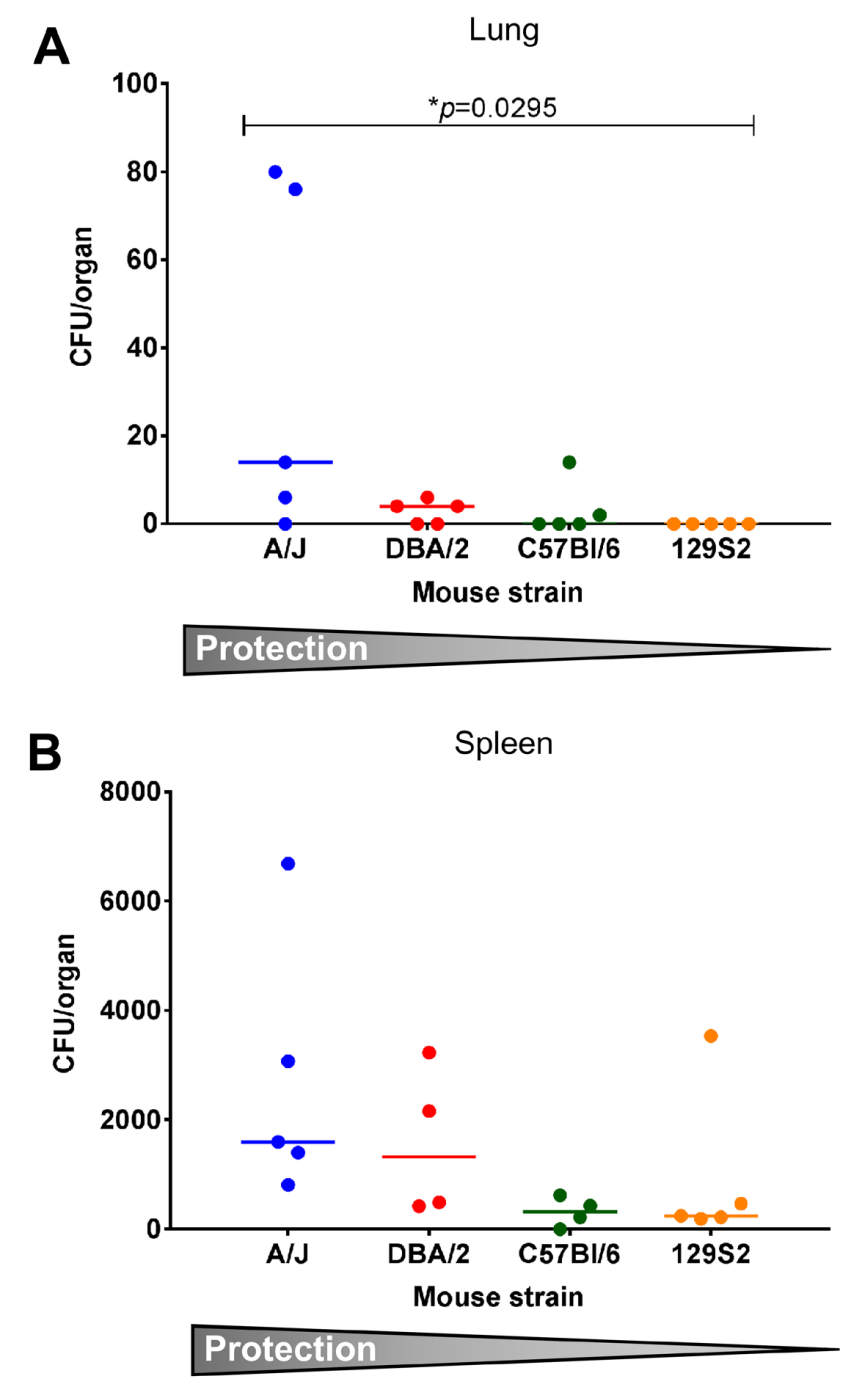
BCG distribution varies between lung and spleen after vaccination of ML high and ML low mice. Approximately half of each lung ( **A**) and spleen ( **B**) was homogenised at the time of Mtb challenge (6 weeks after immunisation) and plated on 7H11 agar plates to determine viable BCG bacteria. Total CFU per organ are reported. Data sets are presented in order of decreasing protection. Each symbol represents one animal. In some instances, bacteria could not be detected and values were set to zero. *p* values were determined by using Kruskal-Wallis test and Dunn’s post test for multiple comparisons. Error bars represent the median and interquartile range. Multiplicity adjusted *p* values are reported. *p*<0.05 was considered statistically significant.

Two independent experiments with separate primary outcomes were carried out to obtain the data described in this report. In Experiment 1, 5 naïve mice of each strain (20 mice total) were culled by anaesthetic overdose, and cardiac blood, lungs and spleens were collected for determination of the ML ratio by flow cytometric analysis (see below for details). The primary outcome for this experiment was the ML ratio ( Figure 1). In Experiment 2, parallel groups of mice (n=5 per group) were BCG-immunised or left untreated. These were allocated at the start of the study to either an immunogenicity group (IFNγ response and BCG dissemination; 10 mice per strain; 40 mice total; Figure 3 and Figure 4), or an *Mtb* challenge group (bacterial burden in lung; 10 mice per strain; 40 mice total; Figure 2). Six weeks after immunisation, mice were infected with *Mtb* or culled for isolation of cells from lung and spleen. In all, a total of 100 mice (25 per strain) was used to obtain all data presented here.

The study was not blinded.

### Vaccination

The BCG Pasteur strain was obtained from Aeras (Rockville, MD, USA) as frozen aliquots. These were stored at -80°C until needed. BCG was then thawed at room temperature and diluted to a final concentration of 2×10 ^6^ CFU/ml in physiological saline solution for irrigation (Baxter Healthcare, Newbury, UK). Each animal received a subcutaneous injection of 100 μl BCG containing 2×10 ^5^ CFU BCG (vaccinated groups). BCG dose was confirmed by plating of an aliquot of the prepared vaccine suspension on 7H11 agar plates. Colonies were counted after 12–14 days of incubation at 37°C. Animals were then rested for 6 weeks before either infection with *Mtb,* or sacrifice for cell isolation.

### BCG enumeration in tissues

Approximately half of each spleen and the right-hand side lobes of the lungs from mice in the immunogenicity group were used to determine the number of viable BCG bacteria in each organ six weeks after vaccination. Tissues were removed aseptically in a microbiological safety cabinet and placed in sterile 2 ml screwcap vials containing 500 μl PBS + 0.05% Tween80 and Precellys 1.4mm ceramic beads (CK14; Peqlab, Sarisbury Green, UK). A Precellys 24 homogeniser (Peqlab) was used to homogenise tissues for 15 s at 5000 rpm before plating. Each entire homogenate was plated onto two 7H11 agar plates containing 10% OADC supplement (Yorlab, York, UK) and 0.5% glycerol. Colonies were counted after 3 weeks of incubation at 37°C.

### Infection with *M. tuberculosis*

Mice were infected intranasally with *M. tuberculosis* Erdman (BEI Resources, Manassas, VA, USA) 6 weeks after BCG immunization and kept in isolators under CL-3 containment. Frozen aliquots of *Mtb* Erdman were thawed at room temperature, and diluted in saline. Mice were anaesthetized by an intraperitoneal injection of a combination of Ketamine (50 mg/kg; Ketalar Pfizer Itd, Kent, UK) and Xylazine (10 mg/kg; Rompun; Berkshire, UK) in saline. Each animal then received 50 μl of the inoculum, estimated to contain 30 CFU. The number of bacteria in the inoculum was confirmed by plating aliquots on 7H11 agar plates containing 10 % OADC and 0.5 % glycerol.

Seven weeks after infection, animals were killed by cervical dislocation. Lungs and spleens were removed aseptically and homogenized by mechanical disruption in sterile PBS, using the plunger of a 5 ml syringe and a 100 μm cell strainer. A series of 10-fold dilutions of tissue homogenates in PBS with 0.05 % Tween 80 were plated onto 7H11 agar plates with 10 % OADC supplement and 0.5 % glycerol. Plates were incubated at 37 °C and colonies counted after 3 weeks.

Protection is expressed by the percent reduction of median CFU/lung in the BCG group compared to the control group for each mouse strain.

### Flow cytometry

Single cell suspensions from spleens were prepared in RPMI-1640 media (Sigma-Aldrich, Dorset, UK) containing 10% heat-inactivated FBS (Labtech International Ltd, Uckfield, UK) and 2 mM L-Glutamine (Fisher Scientific, Loughborough, UK) as soon as possible after sacrifice. Spleens were mechanically disrupted by mashing through a 100μm cell strainer using the rubber end of the plunger from a 5ml syringe. Lungs were collected into RPMI-1640 media without FBS and cut into small pieces of approx. 2mm ^3^ before incubation with 0.5mg/ml Liberase TL (Sigma-Aldrich) and 10μg/ml DNAse (Sigma-Aldrich). The enzyme reaction was stopped by adding an equal amount of media containing 10% FBS and the tissue was mashed as above to obtain single cells. Cells were fixed and red blood cells lysed by adding lyse-fix solution (PhosFlow; Becton Dickinson, Oxford, UK). After fixing, cell suspensions were made up in PBS + 1% FBS. **

Approximately 10 ^6^ cells were stained with the following antibody cocktail in BD Brilliant Stain buffer as per manufacturer’s instructions (Becton Dickinson): CD3-APC/Cy7 (clone 17A2, 1:80), B220-BV510 (clone RA3-6B2, 1:40), Ly6G-BV711 (clone 1A8, 1:40), CD11b-BV650 (clone M1/70, 1:60), CD11c-BV605 (clone N418, 1:40). All antibodies were purchased from Biolegend (via Fisher Scientific).

Cells from each tissue from one mouse per strain were used as fluorescence minus one (FMO) controls. These were stained with the antibody cocktail as described above, but without one of the antibodies. This was done for each antibody. FMO controls were used to guide gating.

OneComp beads (eBioscience via Fisher Scientific, Loughborough, UK) were stained with single antibodies as per manufacturer’s instructions and used to calculate compensation using the automatic function in FlowJo version 10.4. Compensation matrices were manually checked and adjusted where necessary.

Analysis was carried out using FlowJo version 10.4.

The ML ratio was calculated by dividing the percentage of monocytes/macrophages (Mono_Mac) (B220 ^-^ CD3 ^-^ Ly6G ^-^ CD11b ^+^ CD11c ^lo-int^) relative to single cells by the percentage of B cells (B220 ^+^) and T cells (CD3 ^+^): Mono_Mac (% of single cells) / [B cells (% of single cells) + T cells (% of single cells)]

### IFN-γ ELISPOT

To quantify IFN-γ secreting antigen-specific splenocytes, single cell suspensions were prepared by mechanical disruption of the remaining spleen samples from the immunogenicity group through a 100μm cell strainer as soon as possible after sacrifice. After lysis of red blood cells, single cell suspensions were made up in RPMI-1640 media containing 10% heat-inactivated FBS and 2 mM L-Glutamine. 96-well microtiter ELISPOT plates (MAIPS4510, Millipore, Watford, UK) were coated with 10 µg/ml rat anti-mouse IFN-γ (clone AN18, Mabtech, Nacka Strand, Sweden). Free binding sites were blocked with RMPI-1640 supplemented with 10% heat-inactivated FBS and 2 mM L-Glutamine. 2×10 ^5^ of total splenocytes were added and incubated in duplicate with PPD (10 µg/ml), supplemented RPMI as a negative control, or Phorbol myristate acetate (PMA) (0.1 µg/ml, Sigma-Aldrich) and Phytohemagglutinin (PHA) (1 µg/ml, Sigma-Aldrich) as a positive control. After overnight incubation at 37°C in 5% CO _2_, IFN-γ was detected with 1 µg/ml biotin labelled rat anti-mouse antibody (clone R4-6A2, Mabtech) and 1 µg/ml alkaline phosphatase-conjugated streptavidin (Mabtech). The enzyme reaction was developed with BCIP/NBT substrate (5-Bromo-4-chloro-3-indolyl phosphate/Nitro blue tetrazolium) (MP Biochemicals, UK) and stopped by washing the plates with tap water when individual spots could be visually detected (up to 5min). ELISPOT plates were analysed using an automatic plate reader. IFN-γ-specific cells are expressed as number of spot-forming units (SFU) per million spleen cells after non-specific background was subtracted using negative control wells.

### Statistical analysis

Groups of animals were compared, and a *p* value of <0.05 was considered statistically significant. Statistical analysis was carried out using GraphPad Prism version 6. The specific test used for each analysis is described in the figure legends.

## Results

### The ML ratio differs significantly in four inbred mouse strains

Four commercially available inbred mouse strains were chosen to investigate the impact of the baseline ML ratio on BCG vaccine efficacy. A/J, DBA/2, C57Bl/6 and 129S2 mice were selected to represent varying monocyte frequencies and a range of BCG-mediated protection based on available data (Jax Phenome Database https://phenome.jax.org; ^10, 11^). To allow the direct comparison of the ML ratio between strains, animals were age and sex matched and all samples were processed at the same time, stained with aliquots of the same antibody cocktail, and data acquired as a batch. All animals used in this experiment were included in the analysis.

We found significant differences between mouse strains in their baseline ML ratio in blood, spleen, and lung ( Figure 1). Across all tissues, A/J mice showed the lowest ML ratio ( Figure 1 B–D). In spleen and blood, the ML ratio of DBA/2, C57Bl/6 and 129S2 mice was higher than A/J, but only minor differences were found between those three strains ( Figure 1 B and D). In the lung, differences between all strains were more apparent ( Figure 1C). 129S2 mice had the highest ML ratio, with DBA/2 and C57Bl/6 showing intermediate levels. These differences in ML ratio were mostly driven by differing frequencies in monocytes/macrophages, with minor differences in B and T cell frequencies between mouse strains ( Figure. S1–S3).

Flow cytometry raw data This file contains the data underlying the analysis of the ML ratio and cell subsets in blood, spleen, and lung shown in Figure 1, Figure 2B, Figure S1, Figure S2, and Figure S3. Click here for additional data file. Copyright: © 2018 Zelmer A et al. 2018 Data associated with the article are available under the terms of the Creative Commons Zero "No rights reserved" data waiver (CC0 1.0 Public domain dedication).

To assess the impact of these differences in ML ratio on BCG efficacy in our model, we infected mice of all strains with virulent *Mtb* Erdmann.

### Mouse strains with varying ML ratios are differentially protected from *Mtb* infection by BCG

Mice of each strain were vaccinated s.c. with BCG, or left untreated, and infected with *Mtb* Erdmann six weeks later. The extent of protection by BCG was measured by determining the bacterial burden in lung and spleen of vaccinated and control mice ( Figure 2). All mice were immunised with the same inoculum of BCG and infected using the same inoculum of *Mtb*, so that results of the different strains are directly comparable. All animals were included in the analysis and no unexpected adverse events were observed. No more than 10% weight loss was recorded over the duration of the experiment. We found varying degrees of protection ( Figure 2A). A/J mice were the most protected with a difference in median CFU (Δ) between vaccinated and control groups of 1.41 log _10_, followed by DBA/2 (Δ0.81 log _10_) and C57Bl/6 (Δ0.54 log _10_), and 129S2 mice were the least protected (Δ0.37 log _10_). Interestingly, the mouse strain with the highest ML ratio in the lung showed the lowest protection (129S2), and the strain with the lowest ML ratio showed the highest protection (A/J; Figure 2B). Some differences were also observed in the innate susceptibility between strains, most notably C57Bl/6 mice were significantly less susceptible than DBA/2 (Δ1.16 log _10_ CFU) or A/J mice (Δ1.41 log _10_ CFU). Alveolar macrophages play an important role in the defence against respiratory pathogens, however they are not included in the gate used here to quantify monocytes/macrophages ( Figure 2A). While we did not include specific markers for this cell population, they carry a CD11b intermediate (CD11bint), CD11c+ phenotype. We found that the frequency of CD11bint CD11c+ cells was significantly higher in C57Bl/6 mice compared to the other mouse strains ( Figure 2E).

Next, to investigate whether differential BCG-mediated protection from Mtb infection was associated with a differential immune response, we carried out an IFNγ ELISPOT assay.

Mtb challenge CFU raw data This files contains the raw data as colony forming units (CFU) of the graphs in Figure 2. Click here for additional data file. Copyright: © 2018 Zelmer A et al. 2018 Data associated with the article are available under the terms of the Creative Commons Zero "No rights reserved" data waiver (CC0 1.0 Public domain dedication).

### A differential antigen-specific IFNγ response is associated with differential protection

To assess the immune response to mycobacterial antigens in the differentially protected mouse strains, splenocytes from mice vaccinated with BCG were stimulated with PPD, and the number of IFNγ-producing cells was measured by ELISPOT ( Figure 3). IFNγ is a cytokine associated with control of infection in mice ^12^ and reduced risk of TB disease in humans ^6^. We found that there were significantly more antigen-specific IFNγ producing splenocytes in 129S2 mice than in A/J or DBA/2 mice, both of which were better protected by BCG from *Mtb* challenge than 129S2 mice.

Since BCG is a live mycobacterium, and monocytes and macrophages are the natural host cells for these organisms, it was plausible that a high ML ratio and high IFNγ production in 129S2 mice impair BCG survival and dissemination to and/or persistence in tissues, and thus prevent the formation of a protective immune response in the lung.

ELISPOT raw data This file contains the raw data for the graph shown in Figure 3
Copyright: © 2018 Zelmer A et al. 2018 Data associated with the article are available under the terms of the Creative Commons Zero "No rights reserved" data waiver (CC0 1.0 Public domain dedication).

### BCG dissemination to lung and spleen is impaired in ML high mice

To determine the extent to which BCG is found in lungs and spleens after s.c. administration, we plated organ homogenates onto 7H11 agar plates and enumerated viable BCG after 3 weeks of culture ( Figure 4). We generally found low numbers of bacteria per organ, especially in the lung, where the bacterial count did not exceed 80 CFU/lung in any sample. Moderate differences were detected between mouse strains. Most notably, the most protected ML low A/J mice had the highest number of BCG in the lung, while no BCG was detectable in the lung of the least protected ML high 129S2 mice.

These data led us to speculate that certain host immune-environments, such as a high ML ratio, can influence BCG dissemination and/or persistence, which in turn could impact BCG efficacy.

The data presented here are from one experiment as detailed in the Methods section. To ensure reproducibility, we have endeavoured to include as much experimental detail as possible to allow others to repeat the study and confirm the results. Groups of animals of all strains received the same treatments at the same time to minimise technical variation. We are therefore confident that the major differences between strains are true biological differences.

BCG distribution raw data This file contains the raw data as colony forming units (CFU) for the graph shown in Figure 4. Click here for additional data file. Copyright: © 2018 Zelmer A et al. 2018 Data associated with the article are available under the terms of the Creative Commons Zero "No rights reserved" data waiver (CC0 1.0 Public domain dedication).

## Discussion

The varying efficacy of BCG has long been a concern for the control of the TB epidemic. Numerous factors have been discussed as contributors to this, such as population differences, different BCG strains, vaccination schedules, exposure to environmental mycobacteria, co-infections with viruses and/or parasites, and geographical location ^1, 3, 13– 16^. While BCG works in some populations and protects children from pulmonary and extra-pulmonary disease, new vaccines are urgently needed for the populations where BCG fails to protect, such as adolescents and adults in endemic areas ^1, 2^. The incomplete understanding of the mechanisms behind BCG failure, together with limited tools for early assessment of vaccine efficacy, are hampering the development of new vaccines. In order to allow early vaccine testing in a model that better represents the populations most at risk of TB disease, we have taken a correlate of risk observed in human studies and back-translated it into a mouse model. The monocyte to lymphocyte (ML) ratio is a non-specific marker of inflammation and has been shown to be associated with risk of TB disease in several populations, including pregnant HIV infected women, people starting anti-retroviral therapy, BCG vaccinated infants and latently *Mtb* infected adolescents ^7, 8, 17– 19^. Scriba *et al*. show a cascade of inflammatory events as adolescents progress towards disease, which appears to start with increased Type I/II IFN signalling, followed by an increase in monocytes and a decrease in lymphocytes ^19^. An increased risk of TB disease in BCG vaccinated individuals with a high ML ratio indicates that BCG is failing in those populations.

In order to provide proof of principle that the ML ratio, and observations from human studies more generally, can be back-translated to develop more clinically relevant mouse models, we chose to use commercially available, genetically tractable inbred mouse strains. We could show that these have highly varying ML ratios in blood, spleen, and lung, and that these differences, particularly in the lung, were associated with varying BCG-mediated protection from *Mtb* infection. Using mouse strains with genetically different backgrounds means that there are likely other confounding factors that could impact vaccine efficacy. This is exemplified by the DBA/2 mice, which are well protected despite having a relatively high ML ratio across all tissues. This could be due to the fact that they have a higher neutrophil frequency than some of the other strains (data not shown). Although confounding factors may be at play here, this would also be the case in a clinical setting since an altered ML ratio can have different underlying causes. An increased ML ratio is an indicator for inflammation, likely driven by monocytosis during inflammation. Some factors that are thought to influence BCG efficacy also drive inflammation, such as co-infection with viruses or parasites, malnutrition, or exposure to environmental mycobacteria. However, as manipulation of ML ratio *in vitro* impacts on ability to control mycobacterial growth, it is likely that ML ratio itself is contributing to TB risk, independent of the factor driving inflammation ^20^. It is also possible that host genetic factors determine baseline inflammation and ML ratio ^21^. If this is the case in TB endemic populations, inbred mouse strains with naturally varying ML ratios may be a useful model for BCG vaccine variability.

There are genetic differences between the inbred mouse strains used in this study, some of which have been well described, while others are likely to be unknown. Among the most well described genetic factors is the mutation of the interleukin-3 (IL-3) receptor alpha subunit gene in A/J mice, which leads to impaired IL-3 signalling and reduced proliferation of haematopoietic stem cells and their differentiation into myeloid precursors. This is likely to play a role in the low ML ratio observed in these mice ^22, 23^. A/J mice additionally carry a defect in the gene for Naip5, an intracellular pattern recognition receptor involved in control of proliferation of intracellular pathogens ^24, 25^. It may thus have an effect on BCG persistence and dissemination. C57Bl/6 mice carry a polymorphism in the *slc11a1* (formerly *nramp1*) gene, which has been shown to determine susceptibility or resistance to intravenous infection with BCG as defined by BCG load in the spleen ^26^. It does not seem to determine susceptibility to primary *Mtb* infection in mice, and the effect on BCG efficacy is not well studied ^27^. In addition, different inbred mouse strains carry different MHC haplotypes. For the strains used in this study, they are as follows: 129S2, H2 ^b^; C57Bl/6, H2 ^b^; A/J, H2 ^a^; DBA/2, H2 ^d^. Each one confers the ability to recognise a slightly different range of epitopes, although there is overlap. This may also impact BCG- mediated immunity.

It is currently unclear what the relative contributions are of genetic and environmental factors to a change in ML ratio ^21^. Does a naturally high ML ratio in an individual make them more prone to TB disease, or do other factors including *Mtb* itself drive up the ML ratio, causing BCG to fail? Does a naturally high ML ratio in an individual exacerbate an inflammatory response initiated by environmental factors? There is striking heterogeneity in the immune response to BCG in infants vaccinated at birth, including in the ML ratio and cytokine responses ^17^. This points towards an involvement of host factors, as the influence of environmental factors would be limited so early in life. On the other hand, Scriba *et al*. observe increases in inflammation and ML ratio over time in adolescents as an individual progresses towards active TB disease and diagnosis ^19^. This may point towards the pathogen itself as the cause of an altered immune environment.

Interestingly, we found a higher number of antigen-specific IFNγ-producing splenocytes in unprotected 129S2 mice compared to protected A/J mice at the time of *Mtb* challenge. This seemed counterintuitive at first, but is in line with recent findings showing that a sub-population of BCG-vaccinated infants with an increased ML ratio showed increased T cell-mediated cytokine production, and this was associated with increased risk of TB disease ^17^. High monocyte count and pro-inflammatory cytokines are indicators for inflammation. It is possible that BCG efficacy is impaired in inflammatory immune environments, which may be caused by a variety of factors, such as chronic viral infection, age, malnutrition, or prolonged contact with environmental mycobacteria, all factors thought to impact BCG efficacy. Ultimately, BCG is administered into host immune environments with a heterogeneity that is not fully characterised or understood, yet may have implications for its efficacy.

The exact mechanisms behind an altered BCG vaccine efficacy in a ML high immune environment are currently unknown. Our data indicate that impaired dissemination and/or persistence of BCG may play a role in initiating an effective local immune response. A study by Kaveh and colleagues has shown that BCG persists for up to 16 months in lymph nodes and spleen of Balb/c mice and that persistence of live bacilli contributes to optimal protection from *M. bovis* challenge ^28^. Direct investigation of this phenomenon in humans is not possible; there are however reports that indicate that BCG can disseminate and persist for years in a variety of organs in humans ^29, 30^. It is therefore plausible that an immune environment that impairs survival of BCG also impairs vaccine efficacy, although we did not determine in this study whether the absence of live bacilli in lungs of 129S2 mice is due to lack of dissemination or lack of persistence.

The absolute numbers of BCG might be under-estimated in this study as we only enumerated bacilli that were readily growing on standard 7H11 agar plates. Use of PCR methods or culture with resuscitation promoting factors may increase yield and allow for detection of dormant bacilli and their contribution to protection ^31, 32^.

If impaired dissemination and/or persistence in certain immune environments is indeed reducing BCG vaccine efficacy, it is reasonable to assume that other live mycobacterial vaccines would have similar limitations. Variability in the ML ratio and more general heterogeneity in the host immune environment should thus be taken into account when designing new live TB vaccines or vaccines based on boosting BCG.

To circumvent potentially impaired dissemination of parenterally administered live vaccines, mucosal administration of live vaccines could be considered. Recent evidence shows that mucosal administration of BCG confers increased protection in animal models, for example by inducing homing of T cells into protected mucosal niches ^33– 35^. This agrees with the notion that BCG needs to be present in the lungs to initiate a protective immune response in this organ. Furthermore, subunit vaccines should not show decreased efficacy in ML high immune environments if our hypothesis is correct. Both these possibilities will need careful investigation to shed light on the mechanisms behind BCG failure.

We did not investigate the phenotype of monocytes/macrophages or T cells in this study, although it is likely that this plays a role. In particular, inflammatory monocytes might lead to killing of BCG. It will be interesting in future to investigate this, as well as comparing the ML ratio and immune cell phenotypes before and after BCG vaccination in the different mouse strains and locations.

We found a significantly higher proportion of CD11b ^int^ CD11c ^+^ cells in the lungs of C57Bl/6 mice, which are likely to be enriched in alveolar macrophages. Interestingly, C57Bl/6 mice are also the strain least susceptible to Mtb infection without BCG vaccination. While the frequency of this population does not seem to be associated with BCG-mediated protection after subcutaneous administration of the vaccine, it could play a role after intranasal vaccination. Alveolar macrophages are located on the luminal side of the epithelium, therefore encountering antigens and pathogens taken up from environmental air. The monocyte/macrophage population included in our ML ratio on the other hand is likely to be of interstitial nature, residing on the basal side of the epithelium and encountering antigens from the blood stream.

While our data on the susceptibility of naïve mouse strains to *Mtb* infection agrees with other studies ^36^, we found some discrepancies between the data presented here and previously published data on BCG-mediated protection. A study using DBA/2 mice has shown that these mice are not protected by s.c. administration of BCG, but control infection with *Mtb* better when vaccinated i.n. ^11^ This is in contrast to our finding that DBA/2 mice are protected from *Mtb* challenge after s.c. vaccination. However, there are some differences between these two studies that may explain the different findings: a different source of mice was used; protection was assessed at 8 weeks vs 6 weeks after vaccination; the BCG strain and *Mtb* strain differed; and the *Mtb* dose used for challenge was three times higher in the study by Aguilo and colleagues compared to ours. Another report detailing the susceptibility of different mouse strains to *Mtb* challenge shows that A/J mice are notably less protected than C57Bl/6 mice ^10^, while in our hands the opposite is the case. There are important differences between the two studies, including the age and sex of mice used, the time point after vaccination at which protection was measured, and the use of different BCG and *Mtb* strains. In combination, these factors may lead to a differing extent of protection. With particular relevance to this study, the immune cell frequency between male and female animals differs in A/J mice. For example, dataset Donahue5 on the Jackson Laboratory Mouse Phenome Database indicates a higher percentage of monocytes in blood for males compared to females, while T cells are less frequent in males, leading to a higher ML ratio in males ^37^.

In conclusion, the back-translation of a correlate of risk of TB disease, the ML ratio, into an animal model is a step forward for better tools to study the immune mechanisms behind BCG vaccine failure and to test vaccines in a model more relevant to populations most at risk. Hopefully other risk factors can also be back-translated in the future to obtain a more complete picture of vaccine efficacy at very early stages of testing. We would encourage the use of more diverse mouse models in TB vaccine testing and development to reflect the heterogeneity of immune environments found in human populations.

## Data availability

The data referenced by this article are under copyright with the following copyright statement: Copyright: © 2018 Zelmer A et al.

Data associated with the article are available under the terms of the Creative Commons Zero "No rights reserved" data waiver (CC0 1.0 Public domain dedication). 

**Dataset 1**: Flow cytometry raw data – This file contains the data underlying the analysis of the ML ratio and cell subsets in blood, spleen, and lung shown in Figure 1, Figure 2B, Figure S1, Figure S2, and Figure S3. 10.5256/f1000research.14239.d207942 ^38^

**Dataset 2:** Mtb challenge CFU raw data – This files contains the raw data as colony forming units (CFU) of the graphs in Figure 2. 10.5256/f1000research.14239.d197118 ^39^

**Dataset 3:** ELISPOT raw data – This file contains the raw data for the graph shown in Figure 3. 10.5256/f1000research.14239.d197136 ^40^

**Dataset 4:** BCG distribution raw data – This file contains the raw data as colony forming units (CFU) for the graph shown in Figure 4. 10.5256/f1000research.14239.d197137 ^41^

## Supplementary material

**Figure S1: The frequency of T cells varies in tissues of ML high and ML low mouse strains.** T cell (CD3 ^+^) frequencies were determined in blood ( **A**), lung ( **B**) and spleen ( **C**) of four different mouse strains using the gating strategy described in Figure 2A. The ML ratio was calculated using these data. Each symbol represents one animal; box plots represent the median (middle line), 25 ^th^ to 75 ^th^ percentile (box) and minimum to maximum value (error bars). Data sets are presented in order of decreasing protection. *p* values were determined using ordinary ANOVA with Holm-Sidak test for multiple comparisons. Multiplicity adjusted *p* values are reported. A *p* value <0.05 was considered statistically significant.

**Figure S2: The frequency of B cells varies in tissues of ML high and ML low mouse strains.** B cell frequencies (B220 ^+^) were determined in blood ( **A**), lung ( **B**) and spleen ( **C**) of four different mouse strains using the gating strategy described in Figure 2A. The ML ratio was calculated using these data. Each symbol represents one animal; box plots represent the median (middle line), 25 ^th^ to 75 ^th^ percentile (box) and minimum to maximum value (error bars). Data sets are presented in order of decreasing protection. *p* values were determined using ordinary ANOVA with Holm-Sidak test for multiple comparisons. Multiplicity adjusted *p* values are reported. A *p* value <0.05 was considered statistically significant.

**Figure S3: The frequency of monocytes/macrophages varies in tissues of ML high and ML low mouse strains.** Monocyte/macrophage (CD11b ^+^ CD11c ^low-int^) frequencies were determined in blood ( **A**), lung ( **B**) and spleen ( **C**) of four different mouse strains using the gating strategy described in Figure 2A. The ML ratio was calculated using these data. Each symbol represents one animal; box plots represent the median (middle line), 25 ^th^ to 75 ^th^ percentile (box) and minimum to maximum value (error bars). Data sets are presented in order of decreasing protection. *p* values were determined using ordinary ANOVA with Holm-Sidak test for multiple comparisons. Multiplicity adjusted *p* values are reported. A *p* value <0.05 was considered statistically significant.
