# Supplementary figures and images for: High monocyte to lymphocyte ratio is associated with impaired protection after subcutaneous administration of BCG in a mouse model of tuberculosis
Source: F1000Res. 2018 Jun 27;7:296. Originally published 2018 Mar 8. [Version 2] doi: 10.12688/f1000research.14239.2 (PMC6039926; doi:10.12688/f1000research.14239.2)

## Slide 1
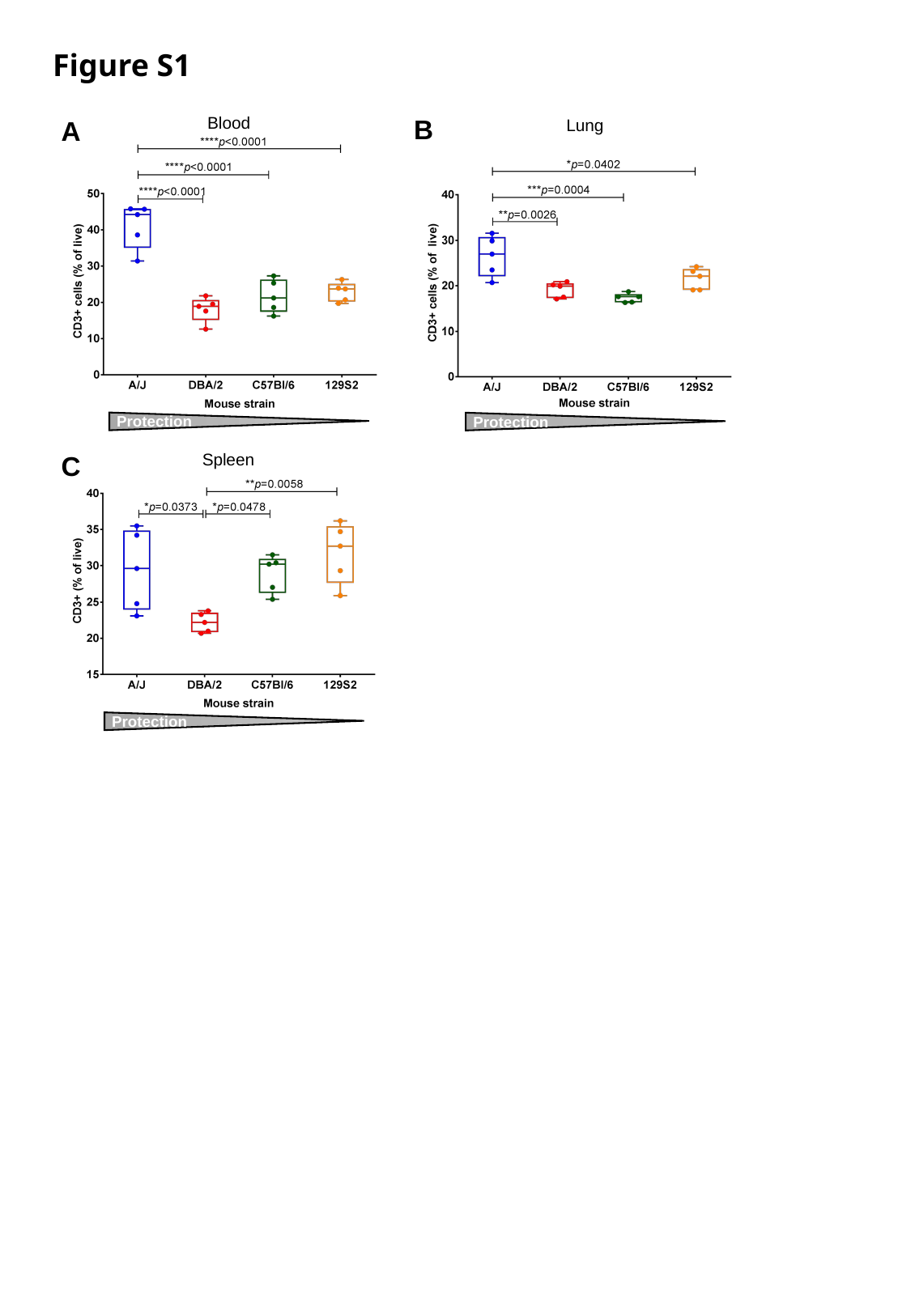

Figure S1
Blood
B
A
Lung
Protection
Protection
Spleen
C
Protection

Supplement: Supplementary file 5 [file f1000research-7-16467-s0004.tgz › 71c06e9f-052b-47c0-9556-335d3597d8c8.pptx]

## Slide 1
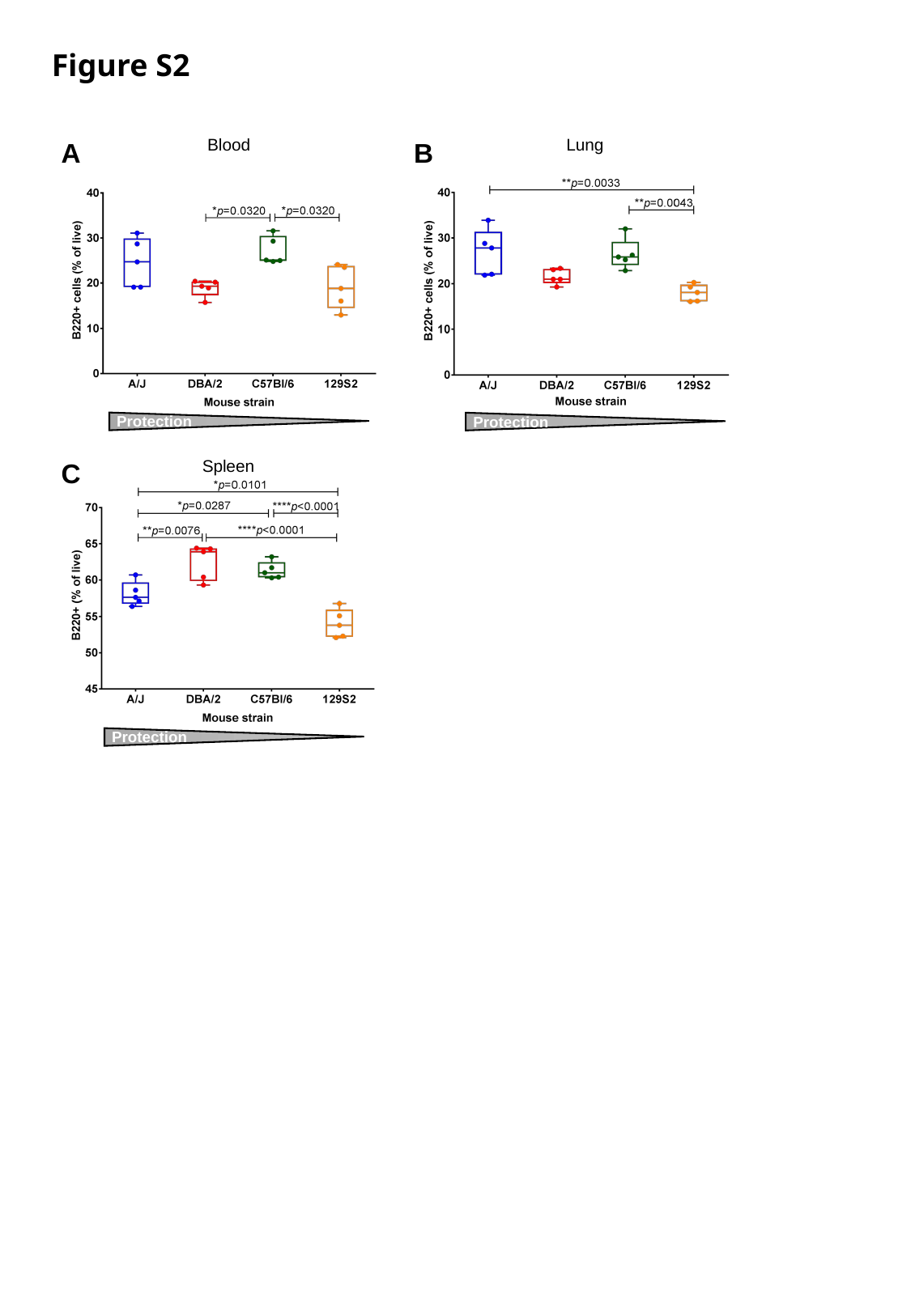

Figure S2
Blood
Lung
A
B
Protection
Protection
Spleen
C
Protection

Supplement: Supplementary file 6 [file f1000research-7-16467-s0005.tgz › 06783da9-23f3-4316-86ec-3f13c31f7fdc.pptx]

## Slide 1
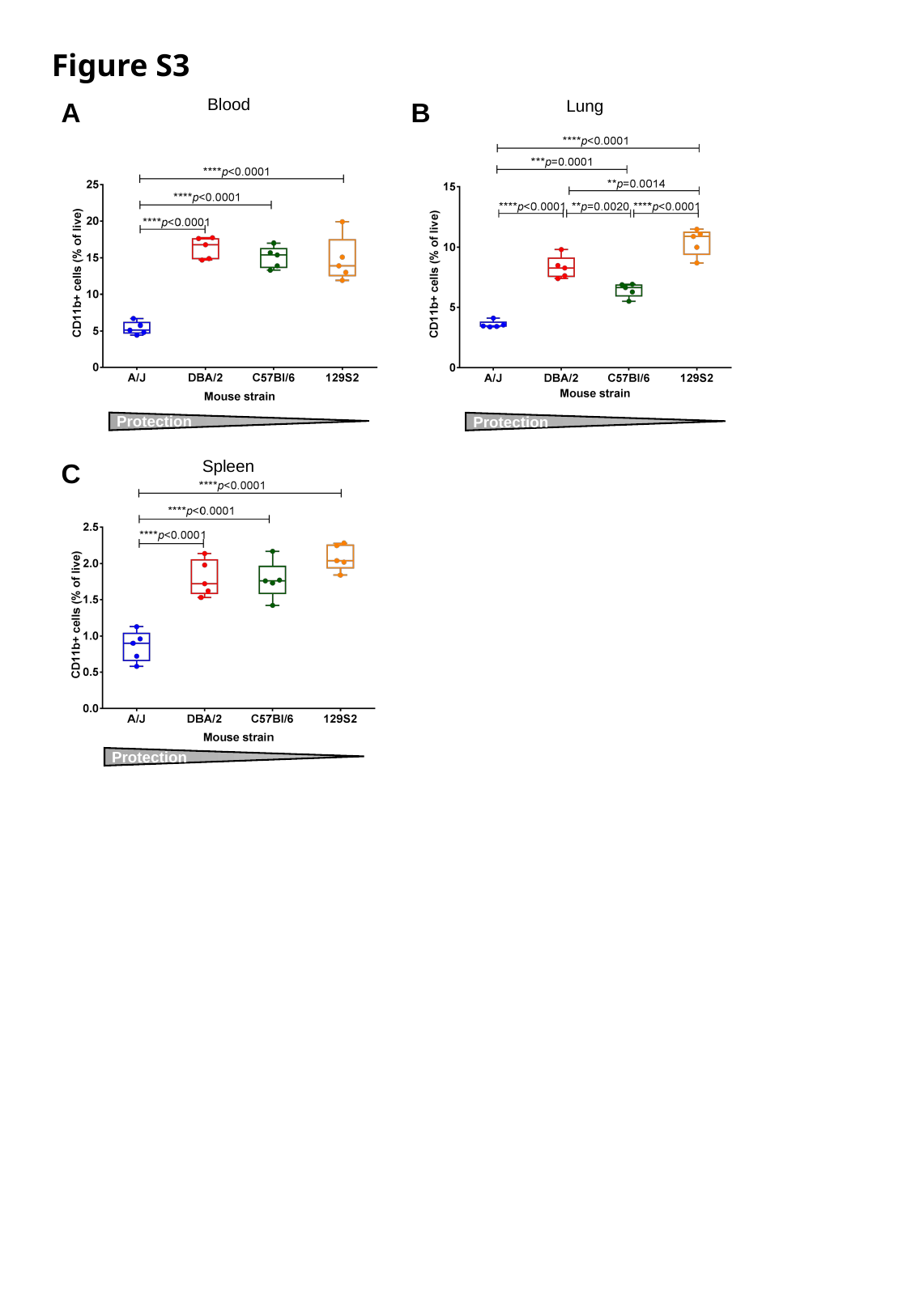

Figure S3
Blood
Lung
A
B
Protection
Protection
Spleen
C
Protection

Supplement: Supplementary file 7 [file f1000research-7-16467-s0006.tgz › ef89c263-f914-47b8-bdaa-f5329ead8fd0.pptx]
