# Supplementary material for: High monocyte to lymphocyte ratio is associated with impaired protection after subcutaneous administration of BCG in a mouse model of tuberculosis
Source: F1000Res. 2018 Jun 27;7:296. Originally published 2018 Mar 8. [Version 2] doi: 10.12688/f1000research.14239.2 (PMC6039926; doi:10.12688/f1000research.14239.2)
Supplement: Peer review history

# Peer review: Referee response for version 2

**DOI:** 10.5256/f1000research.16467.r35522

**Published:** 2018-07-04

**Authors:** Nacho Aguilo

**Affiliations:** 1 Department of Microbiology, Preventive Medicine, and Public Health, University of Zaragoza, Zaragoza, Spain

I am satisfied with the answers given to my comments and therefore I recommend the indexing of the study.

I have read this submission. I believe that I have an appropriate level of expertise to confirm that it is of an acceptable scientific standard.

# Peer review: Referee response for version 1

**DOI:** 10.5256/f1000research.15495.r32475

**Published:** 2018-04-20

**Authors:** Maximiliano G Gutierrez

**Affiliations:** 1 Host-Pathogen Interactions in Tuberculosis Laboratory, Francis Crick Institute, London, USA

The work from Zelmer and collaborators is very interesting, timely, well executed and clearly written. I anticipate a great interest in the tuberculosis vaccine field.

 I have only minor comments and suggestions:

 Regarding introduction and/or discussion, it would be important the authors discuss what are the main genetic differences between the 4 mouse strains selected for this study.

 In Figure 4, it is right to state that colonies vary in tissues but only 2 tissues were analysed here so it would be more appropriate to state "between lung and spleen". Moreover, would it be possible to get the data in lymph nodes as well? Lymph nodes are critical for the responses to vaccination.

 The variation in CFU/organ seem to be higher in animals that have high levels of protection in this study (e.g. A/J mice). Could the authors comment on this? Is it possible to know the ML ratio in the 2 mice showing high CFU/lung from Fig 4A?

 Not sure if the authors have the samples available, but it would have been interesting to see the profiles of Type I/II IFN in tissue at least by qPCR. This information could be of great interest for the tuberculosis field.

 The authors should discuss the limitations of a relatively low number of biological experiments in this work.

I have read this submission. I believe that I have an appropriate level of expertise to confirm that it is of an acceptable scientific standard.

# Response

**Published:** 2018-06-19

**Authors:** Andrea Zelmer

**Affiliations:** LSHTM

Dear Dr Gutierrez

 We would like to thank you for taking the time to review our manuscript, and for the thoughtful feedback provided. We address your individual comments below. 

**The work from Zelmer and collaborators is very interesting, timely, well executed and clearly written. I anticipate a great interest in the tuberculosis vaccine field. I have only minor comments and suggestions:**

**Regarding introduction and/or discussion, it would be important the authors discuss what are the main genetic differences between the 4 mouse strainsselected for this study.**

 We have now included a paragraph in the Discussion to address this.

**In Figure 4, it is right to state that colonies vary in tissues but only 2 tissues were analysed here so it would be more appropriate to state "between lung and spleen". Moreover, would it be possible to get the data in lymph nodes as well? Lymph nodes are critical for the responses to vaccination.**

 We have now specified “lung and spleen” in the legend to Fig. 4 and also in the sub-heading in the results section. 

 It would be very interesting to look at tissue distribution to lymph nodes, and this is a point both reviewers have brought up. Unfortunately, we do not have the samples available and no additional funding is available at the moment to repeat this study. Furthermore, we believe it would be difficult to justify the use of further animals solely for this purpose; however, we will investigate this in future experiments and thank you for the suggestion.

**The variation in CFU/organ seem to be higher in animals that have high levels of protection in this study (e.g. A/J mice). Could the authors comment on this** **? Is it possible to know the ML ratio in the 2 mice showing high CFU/lung from Fig 4A?**

 Since a different set of animals was used for flow cytometry and BCG distribution studies (outlined in the Methods section), it is not possible to directly compare the ML ratio and BCG CFU of individual animals.

 With regards to variation in Fig. 4, we believe this is likely to be a technical effect – we detected very few bacilli in general, and especially in the lung, we did not detect any bacteria in some samples from lesser protected animals. This does not mean there are non – it just means that we were not able to detect them with the method used. As such, it is difficult to interpret the real variation within those mouse strains.

**Not sure if the authors have the samples available, but it would have been interesting to see the profiles of Type I/II IFN in tissue at least by qPCR. This information could be of great interest for the tuberculosis field.**

 Unfortunately we do not have those samples available. It is an important point, and would indeed be very relevant. We plan to investigate this in future studies.

**The authors should discuss the limitations of a relatively low number of biological experiments in this work.**

 We have added a comment to this effect at the end of the results section.

# Peer review: Referee response for version 1

**DOI:** 10.5256/f1000research.15495.r32536

**Published:** 2018-04-09

**Authors:** Nacho Aguilo

**Affiliations:** 1 Department of Microbiology, Preventive Medicine, and Public Health, University of Zaragoza, Zaragoza, Spain

The study by Zelmer *et al.* evaluates in different mouse genetic backgrounds whether the ratio of monocytes/macrophages vs T lymphocytes could affect BCG-induced protection. This study results are highly interesting as it evaluates in an animal model data derived from a previous vaccine efficacy trial. In addition, it highlights a critical question in the field of vaccine evaluation in mice, which is the genetic heterogeneity among inbred mouse strains. This issue is of particular importance when we are assessing vaccines which aim to be moved to humans. During the reading of the manuscript I have found some minor questions that I think should be amended to improve the quality of the study.

 1. I think that the gating criteria used to enclose the monocyte/macrophage population (CD11b ^+^CD11c ^int ^cells) is excluding myeloid populations that could be highly informative for the outcome of the study. This is particularly important in the case of the lungs, where naïve alveolar macrophages have a phenotype CD11b ^int^CD11c ^+^. Alveolar macrophages represent a first-line defense against respiratory pathogens, and therefore results could be very informative to know which is the status of these cells in the different strains used. More even if we consider that authors compare directly data from BCG-induced protection with the ML ratio in the lungs (Figure 2B).

 2. I suggest to make the comparison between ML ratio and protection using a linear regression, so statistical significance of the shown data could be assessed.

 3. I find highly interesting the data showing the differences in BCG persistence among the four strains, more even considering the influence of vaccine persistence in protective efficacy. This result evidences that live vaccine persistence does not only depends on the vaccine itself, but also on the host, and this is a question that should be considered in the future to study novel tuberculosis vaccines. My question is whether the authors could provide some data of BCG persistence in the local draining lymph nodes (in addition to lungs and spleen), as they are the primary sites of BCG biodistribution following subcutaneous/intradermal vaccination.

I have read this submission. I believe that I have an appropriate level of expertise to confirm that it is of an acceptable scientific standard, however I have significant reservations, as outlined above.

# Response

**Published:** 2018-06-19

**Authors:** Andrea Zelmer

**Affiliations:** LSHTM

Dear Dr Aguilo

 Thank you for reviewing our manuscript, and for providing timely and helpful comments and feedback. We discuss your individual points below.

**The study by Zelmer *et al.* evaluates in different mouse genetic backgrounds whether the ratio of monocytes/macrophages vs T lymphocytes could affect BCG-induced protection. This study results are highly interesting as it evaluates in an animal model data derived from a previous vaccine efficacy trial. In addition, it highlights a critical question in the field of vaccine evaluation in mice, which is the genetic heterogeneity among inbred mouse strains. This issue is of particular importance when we are assessing vaccines which aim to be moved to humans. During the reading of the manuscript I have found some minor questions that I think should be amended to improve the quality of the study.**

**1. I think that the gating criteria used to enclose the monocyte/macrophage population (CD11b ^+^CD11c ^int ^cells) is excluding myeloid populations that could be highly informative for the outcome of the study. This is particularly important in the case of the lungs, where naïve alveolar macrophages have a phenotype CD11b ^int^CD11c ^+^. Alveolar macrophages represent a first-line defense against respiratory pathogens, and therefore results could be very informative to know which is the status of these cells in the different strains used. More even if we consider that authors compare directly data from BCG-induced protection with the ML ratio in the lungs (Figure 2B).**

 We have analysed the frequency of CD11b ^int^CD11c ^+^cells in the lungs of the four different mouse strains, and have added Fig. 1E, as well as described the data in the Results section and discussed it in the Discussion. In brief, the frequency of this population is significantly higher in C57Bl/6 mice, and seems to be associated with susceptibility of naïve mice, but not BCG-mediated protection.

**2. I suggest to make the comparison between ML ratio and protection using a linear regression, so statistical significance of the shown data could be assessed.**

 This would indeed be an informative analysis; however, our experimental design does not allow it here. Since the data shown from flow cytometry and protection studies were acquired from two different sets of mice (see Methods section), the data are not paired, and it would not be appropriate to link them in a linear regression.

**3. I find highly interesting the data showing the differences in BCG persistence among the four strains, more even considering the influence of vaccine persistence in protective efficacy. This result evidences that live vaccine persistence does not only depends on the vaccine itself, but also on the host, and this is a question that should be considered in the future to study novel tuberculosis vaccines. My question is whether the authors could provide some data of BCG persistence in the local draining lymph nodes (in addition to lungs and spleen), as they are the primary sites of BCG biodistribution following subcutaneous/intradermal vaccination.**

 This is an important point, and was also raised by Dr Gutierrez, the other reviewer. As you will see from our response to Dr Gutierrez’ comments, we unfortunately do not have the samples available and no additional funding is available at the moment to repeat this study. Furthermore, we believe it would be difficult to justify the use of further animals solely for this purpose; however, we are very interested to investigate the role of BCG distribution in vaccine efficacy in more detail in the future, and thank you for the suggestion.
